# Preparation and Carbon-Dependent Supercapacitive Behaviour of Nanohybrid Materials between Polyoxometalate and Porous Carbon Derived from Zeolitic Templates

**DOI:** 10.3390/ma13010081

**Published:** 2019-12-22

**Authors:** Heng Wang, Takeshi Shimizu, Hirofumi Yoshikawa

**Affiliations:** 1 School of Material and Chemical Engineering, Zhengzhou University of Light Industry, Zhengzhou 450002, China; wangheng@zzuli.edu.cn; 2 School of Science and Technology, Kwansei Gakuin University, 2-1 Gakuen, Sanda, Hyogo 669-1337, Japan; dzh92101@kwansei.ac.jp

**Keywords:** nanohybrid, supercapacitor, porous carbon, zeolitic, polyoxometalate

## Abstract

An electrochemical cell combining the energy storage characteristics of the chemical redox reaction and a physical capacitor effect presents advantages including high energy and power densities, and long durability. In this study, we prepared nanohybrid materials between polyoxometalate (POM) and porous carbon, which have different porous structures and pore sizes, using different zeolitic templates. The POM molecules were loaded inside the porous carbon, and these POM/carbon nanohybrid materials were used as cathode active materials for lithium–ion batteries (LIBs). The performance of these molecular cluster batteries (MCBs) was significantly dependent on the porous carbon. Operando X-ray absorption fine structure (XAFS) and ^7^Li solid-state nuclear magnetic resonance (NMR) measurements of the POM/carbon-MCBs revealed that three-dimensional porous carbon with high surface areas can improve the performance. The results highlight the remarkable performance of porous carbon with a three-dimensionally-linked pore network structure as an additive for supercapacitors to realise high-performance energy storage devices.

## 1. Introduction

The development of energy storage systems with high energy and power density and good cycle stability, has become an important task worldwide, owing to the increasing demand for environmental benignity, as well as the urgent need for a clean and sustainable energy resource [1,2,3,4,5,6]. Capacitors and rechargeable batteries, which operate via different mechanisms, have been widely studied as energy storage devices [7,8,9]. Rechargeable batteries have a high energy density, but a low power density and poor cycling stability, thereby rendering them unsuitable for prolonged application. Capacitors, on the other hand, which store electrostatic energy through the formation of electrical double layers (EDLs) at electrode interfaces, have a long life and higher power density; however, their energy density is relatively low. A promising solution is to develop novel hybrid energy storage systems, based on the advantages of a chemical redox reaction and a physical capacitor effect, which can achieve both high energy and power densities [10,11,12,13].

Nanocarbons, such as single-walled carbon nanotubes (SWNTs) and templated mesoporous carbons, have long diffusion paths and large pore sizes for rapid ion movement in charge/discharge processes [14,15,16,17]. However, exceedingly large pore sizes significantly lower the electrode density, thereby drastically decreasing the volumetric capacitance and resulting in a low volumetric energy density. To avoid this issue, the pore size should be as small as possible, while retaining fast ion transport. It is possible to meet these two requirements with zeolite-templated carbon (ZTC), an ordered microporous carbon obtained as a negative replica of a zeolite template with narrow pore size distributions in the mesopore range (>2 nm) [18,19,20,21].

Polyoxometalates (POMs) are well established to achieve a high capacity for energy storage applications (LIBs and capacitors) due to their stable multielectron redox behaviour and structural diversity [21,22,23,24,25]. Theoretical and experimental studies of molecular cluster batteries (MCBs), in which a traditional Keggin-type POM, [PMo_12_O_40_]^3−^, was used as their cathode active materials, revealed that POM showed an ‘electron sponge’ behaviour based on its large electron uptake number during discharge when used as the cathode of LIBs [26,27,28,29]. Such electron sponge behaviour indicates that POMs are promising cathode active materials for high-performance rechargeable batteries. Thus, electrode materials with both of the characteristics of the battery and the capacitor, termed ‘redox capacitor’ (supercapacitor), could be obtained by a combination of both advantages of ZTC and POMs [21,22]. Thus far, we have reported such supercapacitance behaviour of nanohybrid materials between POM and nanocarbons such as SWNTs and graphenes, and found that their higher capacities due to the electrical double layer capacitance (EDLC) of nanocarbons is enhanced by the nanohybridisation with POM [30,31]. Hence, to achieve higher supercapacitances, the key is to use high-surface-area nanocarbons, such as nanoporous carbons, which show higher EDLCs. 

**Scheme 1:**
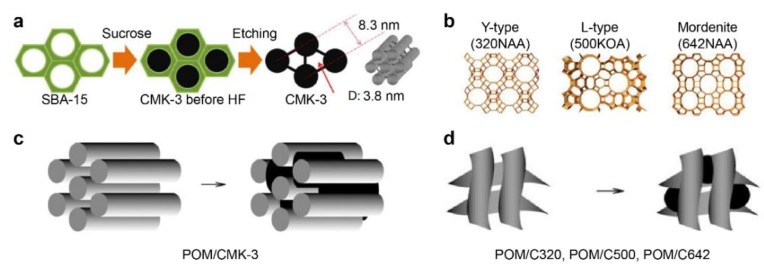
(**a**) Preparation of CMK-3. (**b**) Structure of 320NAA, 500KOA, and 64NAA.: (**c**) Hybrid material between CMK-3 and Keggin-type polyoxometalate (POM/CMK-3), (**d**) Hybrid material between C320, C500, C642 and Keggin-type polyoxometalate (POM/C320, POM/C500, POM/C642).

In this study, we prepared nanohybrid materials between POM clusters and porous carbons derived from zeolitic templates: CMK-3, C320, C500 and C642 (Scheme 1), with different porous structures, and examined their performance as cathode active materials for MCBs. We also carried out operando XAFS, and ^7^Li solid-state NMR analyses for MCBs of the POM/carbon explored the contribution of carbon in these nanohybrid materials. 

## 2. Materials and Methods 

### 2.1. Preparation of POM, SBA-15, CMK-3, C320, C500, and C642

Keggin-type polyoxometalate (POM), TBA_3_[PMo_12_O_40_] (TBA=[N(CH_2_CH_2_CH_2_CH_3_)_4_]^+^), was synthesized according to the previously reported method [32].

All the chemicals reagents used in our experiments were used as received without further purification. A template for carbon, SBA-15 (mesoporous silica) (99%), was prepared using a typical procedure. In this study, 3.5 g (0.603 mmol) of P123 (poly(ethylene glycol)-block-poly (propylene glycol)-block-poly(ethylene glycol)) (Aldrich) was dissolved in 130 mL (0.212 mol) of HCl (Wako) under magnetic stirring [33]. 

After dissolution of the surfactant, 7.7 g (37 mmol) of tetraethylorthosilicate (TEOS) (Wako) (98%) was added into the initial solution. Then, the mixture was heated at 35 °C and maintained under vigorous stirring for 24 h. After stirring at 100 °C for 24 h for aging, the products were filtrated, washed with distilled water, and dried at 70 °C for 6 h. Finally, the as-synthesised samples were calcined for 5 h at 500 °C under air to remove the template.

CMK-3 was then prepared using SBA-15 as a template according to the literature [34]. For this, 1.0 g of SBA-15 was added to a solution of 1.25 g sucrose (Wako) as a carbon source and 0.14 g sulphuric acid dissolved in 5.0 mL of water. The solution was heated at 100 °C for 6 h, following which the temperature was raised to 160 °C, at which dehydration and polymerisation of the sucrose occurred over a period of 6 h. After repeating the soaking–heating cycle, the solid was heated at 900 °C for 6 h under N_2_ flow. The temperature was raised by 5 °C/min. SBA-15 was removed by dissolution at room temperature for 48 h with HF solution (46% HF: 10 mL, water: 60 mL). Finally, CMK-3 was washed with water and dried at 85 °C under vacuum for 12 h. 

Similarly, C320, C500 and C642 were prepared using Y-type zeolite 320NAA (Tosoh), L-type zeolite 500KOA (Tosoh) and mordenite zeolite 642NAA (Tosoh) instead of SBA-15.

### 2.2. Preparation of POM/CMK-3, POM/C320, POM/C500, and POM/C642 Nanohybrid Materials

To graft the POM molecules onto the surfaces of CMK-3 with a weight ratio of 1:2, we used the same method as for our previous polyoxometalate/single-walled carbon nanotubes (POM/SWNTs) or POM/graphene nanohybrid materials; an acetonitrile solution (5 mL) of TBA_3_[PMo_12_O_40_] (50 mg, 20 mmol) was added to a toluene suspension (150 mL) of the as-prepared CMK-3 (100 mg) under vigorous stirring at room temperature. After stirring until the turbidity disappeared, the solution was filtrated using a polytetrafluoroethylene (PTFE) filter with a pore diameter of 0.2 μm. Finally, the precipitation was washed with toluene and dried at 70 °C in a vacuum. Similarly, POM/C320, POM/C500 and POM/C642 nanohybrid materials with a similar ratio (1:2) were obtained by using C320, C500 and C642, respectively, instead of CMK-3. 

### 2.3. Characterisation

The materials were characterised by infrared (IR) spectroscopy (Spectrum One, PerkinElmer). The morphologies of the materials were characterised by scanning electron microscopy (SEM, JEOL JCM-6000) and transmission electron microscopy (TEM, JEM-2100F/JEOL). Nitrogen adsorption/desorption isotherms were acquired by using BELSORP-max. The specific surface area was estimated by the Brunauer–Emmett–Teller (BET) method. The Broekhoff de Boer (BdB) method was applied to the adsorption curves to evaluate the average pore size distributions. 

### 2.4. Electrochemical Measurements of Carbon Material and Nanohybrid Materials

The POM/carbon nanohybrid material was used as a cathode active material for MCBs to investigate the battery performance. The cathode was prepared by mixing this nanohybrid material, carbon black and polyvinylidenefluoride (PVDF) at a weight ratio of 3:5:2. The resulting slurry was spread evenly onto an aluminium foil, and dried under vacuum overnight at 105 °C. The cathodes used here had a mass loading of ca. 10 mg cm^−2^. A lithium foil was used as the anode. The electrolyte was a solution of 1 M LiPF_6_ in ethylene carbonate (EC)/diethyl carbonate (DEC) (1:1, in weight). Coin cell batteries were fabricated in an Ar atmosphere. The charge/discharge measurements were carried out in the voltage range of 1.5–4.2 V on a Hokuto HJ1001-SM8A, with a constant current of *I* = 1.0 mA. The carbon material was also prepared into cathodes, using the same procedure and a 4:1 weight ratio of carbon material/PVDF, following which their capacitance behaviour was measured.

### 2.5. Operando XAFS Measurements

For operando XAFS measurements, the battery was fabricated in a similar manner, using a special battery cell with a Kapton film as an X-ray window. Operando Mo *K*-edge XAFS measurements were carried out in the transmission mode using the BL-NW10A beam lines at Photon Factory. During the charge/discharge reactions, a quick XAFS (QXAFS) recording mode was used to obtain Mo *K*-edge XAFS spectra, which were recorded for 2 min/spectrum with an interval of 1 min. Mo metal, MoO_2_ and POM diluted with boron nitride, were used as standard materials to analyse the relationship between the absorption edge energy of the Mo *K*-edge and the averaged valence of Mo. The XAFS spectra were analysed using the software REX2000 (Rigaku Corp).

### 2.6. Operando Solid-State NMR Spectroscopy

A pellet-type cathode with a weight ratio of POM/C320 nanohybrid materials: carbon black: PVDF = 1:7:2 was prepared with a thickness of 0.5 mm for in situ nuclear magnetic resonance (NMR) experiments. The prepared cathode was placed into an extremely small custom-made quartz cell (18 mm × 2.8 mm × 2.8 mm) with a glass fibre separator (Whatman, glass microfiber filters), and an anode was a lithium metal foil. Before sealing the cell with glue, the electrolyte (DEC:EC = 1:1 with 1 M LiPF_6_) (0.1 mL) was added. The battery was fabricated in a glove box. Pt foil current collectors on both sides were connected to a charge/discharge device with copper wires.

In situ ^7^Li NMR of this electrochemical cell was conducted on a JEOL-ECA 700 solid state NMR spectrometer operating at a ^7^Li NMR frequency of 38.87 MHz with repeated single pulse excitation and a relaxation delay of 0.8 s. A total of 1280 scans was collected for each spectrum during the electrochemical measurements. The ^7^Li chemical shifts were externally referenced to solid LiCl at 0 ppm. Charge/discharge measurements were performed in the voltage range of 1.3–4.2 V at a constant current of 0.1 mA. 

## 3. Results and Discussion

### 3.1. Synthesis of POM/CMK-3, POM/C320, POM/C500, and POM/C642

**Figure 1:**
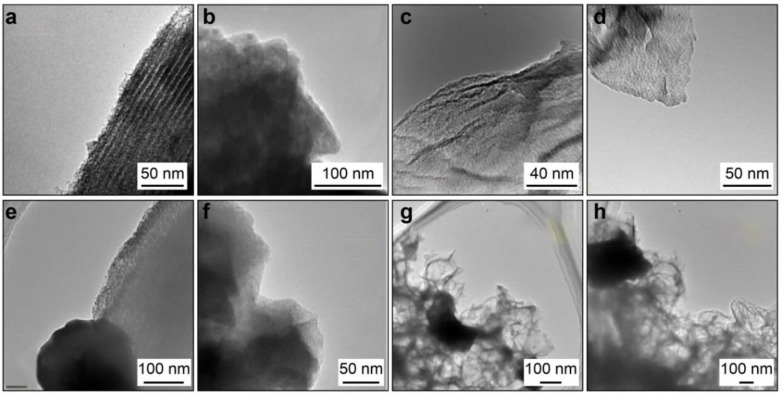
Transmission electron microscopy (TEM) images of (**a**) CMK-3, (**b**) C320, (**c**) C500, (**d**) C642, (**e**) POM/CMK-3, (**f**) POM/C320, (**g**) POM/C500, and (**h**) POM/C642.

Transmission electron microscopy (TEM) images of the as-prepared porous carbon and POM/carbon nanohybrid materials are shown in Figure 1. In Figure 1a, the parallel lines are due to the mesostructure of CMK-3 where the carbon rods are fixed in a parallel position and form a *P*6mm hexagonal lattice. Figure 1b–d also show micrographs of the other as-prepared porous carbons with a smooth surface. Figure 1e–h show the aggregated POM molecules attached to the surfaces of the porous carbons. Figure S1 shows the energy-dispersive X-ray (EDX) spectra in the area of transmission electron microscopy (TEM) image (Figure 1f). It is confirmed as to the presence of molybdenum as well as carbon; the copper peaks are ascribed to the TEM grids. The IR spectra for the POM/carbon nanohybrid materials are shown in Figure S2. They indicate that [PMo_12_O_40_]^3−^ anions are present without any structural changes from the original one.

**Figure 2:**
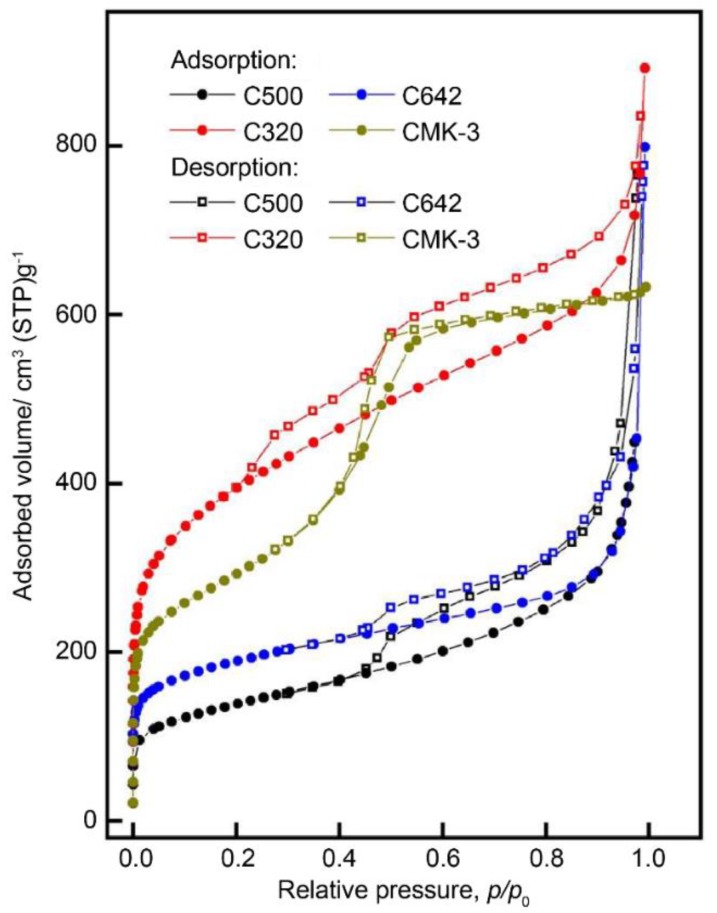
Nitrogen adsorption–desorption isotherms of mesoporous carbon: CMK-3, C320, C500, and C642.

The nitrogen adsorption–desorption isotherms and pore size distributions of the mesoporous carbon CMK-3, C320, C500 and C642 are depicted in Figure 2. The pore sizes were calculated from the adsorption curve of the isotherms. As shown in Figure 2, CMK-3 and C320 have high N_2_ adsorption. The C320 adsorption/desorption isotherm exhibits the largest hysteresis loop among these four materials, implying that C320 has a mesopore and may arise from the strong chemical interaction of the pore wall with N_2_; on the other hand, the isotherms of CMK-3 are in good agreement with those previously reported [35]. The abrupt increase in adsorption around p/p_0_ = 0.5 for CMK-3 implies blockage of the mesopores. On the other hand, the adsorption curves for C320, C500 and C642 smoothly increase. This difference probably arises from the porous structure of CMK-3, which is an ensemble of straight channels in contrast to the three dimensional (3D) porous structure of C320, C500 and C642, where clogging hardly occurs. The BET specific surface area, structure and pore size of the porous carbons are summarised in Table 1. Although it is considered that the properties are dependent on the silica and each zeolite template structure, C320 exhibits a 3D mesoporous structure with the highest surface area among these materials. 

### 3.2. Electrochemical Performances of POM/CMK-3, POM/C320, POM/C500, and POM/C642

We examined the specific capacitances for the porous carbon materials, CMK-3, C320, C500 and C642 before testing the performance of the POM/carbon nanohybrid materials. Figure S3 shows the first ten charge–discharge curves, in which the capacities are normalised by the unit weight of each carbon material. The discharge curves for the four batteries exhibit gradual voltage decreases. The capacities are constantly at approximately 70 Ah/kg for C320, 55 Ah/kg for CMK-3, and 62 Ah/kg for C500, whereas those for C642 remain over 38 Ah/kg. Although only the surface areas do not govern the capacitances, C320 has the largest surface area and also exhibited the highest capacitance. 

**Figure 3:**
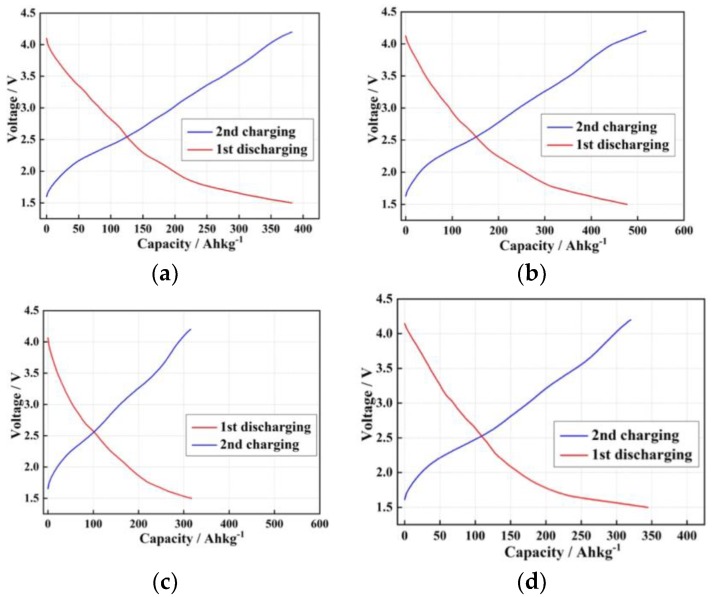

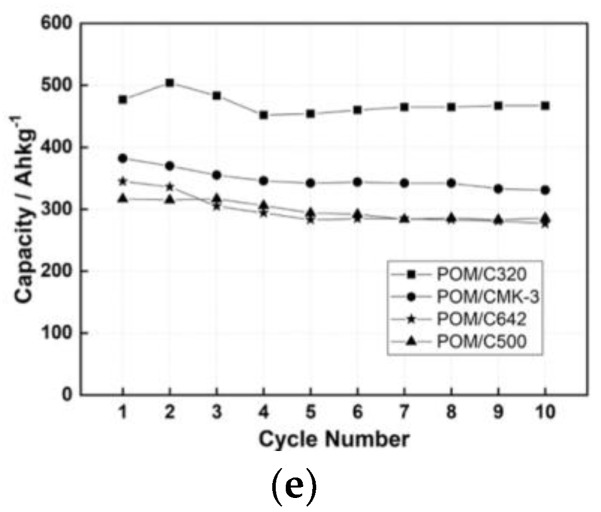
First discharge and second charge curves in the voltage range of 1.5–4.2 V with a constant current of *I* = 1.0 mA for (**a**) POM/CMK-3, (**b**) POM/C320, (**c**) POM/C500, (**d**) POM/C642. (**e**) Cycle performance for POM/CMK-3, POM/C320, POM/C500, and POM/C642.

Figure 3a–d show the first discharge and second charge curves for the MCBs of POM/CMK-3, POM/C320, POM/C500 and POM/C642, where the capacities are defined as those per unit weight of POM in the nanohybrid materials, corresponding to 10 wt % of the cathode. Although the POM content in the cathode is small since POM is an insulator, it was used to compare with our previous results on nanohybrid materials. The discharge and charge curves were shown by red and blue lines in Figure 3, respectively. All the charge/discharge curves in the initial 10 cycles for four kinds of MCBs are shown in Figure S4. As shown in Figure S4, the discharge curves for these four batteries exhibit gradual voltage decreases with different capacities: 377, 489, 315 and 349 Ah/kg for MCBs of POM/CMK-3, POM/C320, POM/C500 and POM/C642, respectively. 

Figure 3e shows the cycle performances of these discharge capacities during ten cycles, where there’s no significant capacity decrease in each material. It is concluded that the POM/C320-MCB stably exhibits a higher capacity than MCBs of nanohybrid materials using the other three porous carbons. The capacity of POM/C320 is also higher than those of POM/SWNT, POM/graphene and only POM (previously reported by us). In EDL, ions, before adsorption, need to be transferred from a bulk solution to the surface of the electrode through narrow pores. Therefore, decreasing the ion-transfer resistance in the pores is vital to realising a high-power density EDLC. POM/C320, with its three-dimensional structure, higher surface area, suitable pore size and efficient ion diffusion paths, stably enhances the capacitor effect. 

Additionally, we also tested the battery performances using the cathodes including 50 and 70 wt % POM/C320 nanohybrid materials. As shown in Figure S5, these capacities significantly decreased when the content of nanohybrid materials in the cathode was increased, due to the insulating characteristics of POM. Although the high contents of active materials are important for practical use, the low content was used to discuss their fundamental nature of energy storage in this work. This is probably reasonable since the used conductive carbon, carbon black, has low capacities (Figure S6).

### 3.3. Operando XANES Analyses

**Figure 4:**
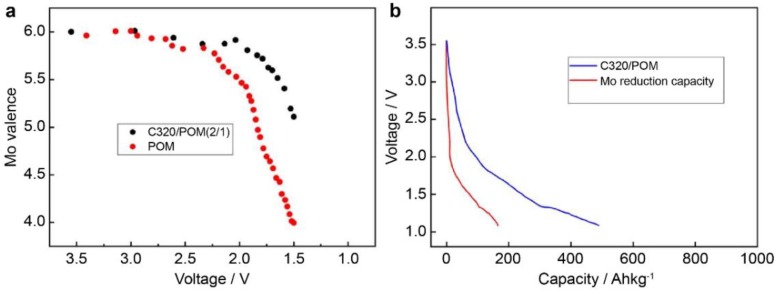
(**a**) Averaged Mo valence of the polyoxometalate (POM) molecule in the microcrystal POM-MCBs (red) and in the POM/C320-MCBs (black) as a function of the cell voltage. (**b**) Experimental discharge curve capacity (blue) and calculated curve (red) from the valence change in POM, for the POM/C320-MCB.

In order to understand this higher capacity of the POM/C320 MCB, operando Mo *K*-edge XAFS measurements were carried out during the charge/discharge processes of the POM/C320 MCB. The red circles in Figure 4a show the averaged valence *N*_v_ of the Mo ions in this MCB during discharge, which were estimated from the edge energies of the Mo *K*-edge X-ray absorption near-edge structure (XANES) spectra (Figure S7). Figure 4a also shows the data for the microcrystal POM-MCB (black circles), which were obtained previously. The values of *N*_v_ for this MCB show a decrease from 6.0 to 4.0 in the voltage range of 3.5–1.5 V in discharge. This change in *N*_v_ by 2.0 implies that all the 12 Mo^6+^ ions in POM are reduced to Mo^4+^; namely, one POM molecule can store 24 electrons. Note, though, that the battery capacity calculated from this value is ca. 260 Ah/kg. Contrary, the POM/C320 MCB exhibits a much smaller change in *N*_v_; the initial value of 6.0 decreases to 5.1 at 1.5 V, indicating that the POM molecules are not fully reduced at this voltage. Although the battery capacity in the range of 4.0–1.5 V for the POM/C320-MCB is higher than the microcrystal POM-MCB (ca. 265 Ah/kg), the contribution from the chemical redox change in POM is smaller as suggested by XANES analyses. This implies that the increased capacity for the POM/C320 MCB is derived from the significant increase in the capacitor effects, which overcome the decrease in the chemical redox capacity. In addition, the insufficient reduction in the POM/ C320-MCB suggests an interaction between POM and C320 in the nanohybrid materials, which decreases the reduction potential of POM as reported previously in POM/graphene nanohybrid materials [31]. 

Using the values of *N*_v_ as a function of the battery voltage (Figure 4a), we calculated the theoretical discharge curve for the POM/C320-MCB, which is caused by only the chemical redox reaction of POM. The results are shown as the red curve in Figure 4b, and are compared with the experimental second discharge curve for the POM/ C320-MCB (plotted as a blue line in Figure 4b). There is a significant difference between the two curves throughout the voltage range of 4.0–1.5 V, which could be ascribed to the contribution from the capacitor effect due to the nanohybridisation between POM and C320. The weight of C320 is two-thirds that of the hybrid materials. The capacity of POM/C320, except that caused by the chemical reduction of POM, becomes ca. 340 Ah/kg. This value can be normalised as ca. 170 Ah/kg per weight of C320 in the nanohybrid materials, and is much higher than the capacitance of C320 (70 Ah/kg). This indicates that the presence of the POM molecules on the surfaces of C320 should greatly enhance the capacitor effect of C320. That is, more Li^+^ ions are adsorbed onto the surface of the nanocarbon due to electrostatic interactions between the POM anion and Li^+^ cation in the interfaces. 

### 3.4. Operando Solid-State ^7^Li NMR Analyses

**Figure 5:**
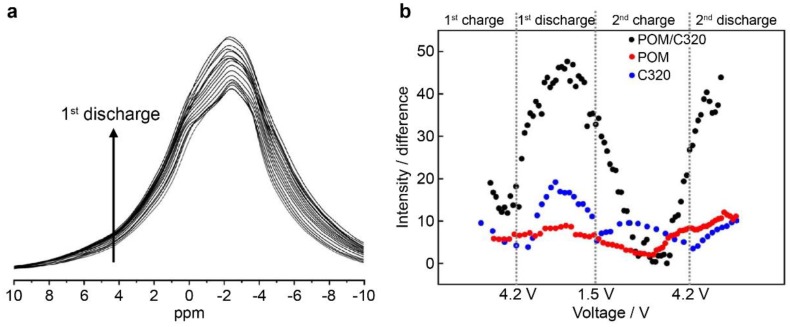
(**a**) ^7^Li nuclear magnetic resonance (NMR) spectra around 0 ppm evolution during the first discharge. (**b**) Intensity evolution of Li^+^ around POM resonance of POM/C320, POM, and C320.

Operando solid-state ^7^Li NMR measurements were carried out to study the capacitor effects in the POM/C320-MCBs, because ^7^Li NMR can probe the structural and dynamic aspects of Li^+^ and its interactions with paramagnetic species [36,37,38]. The overall, solid-state ^7^Li NMR spectrum of the POM/C320-MCBs during charge/discharge (Figure S8a) is shown in Figure S8b. Two ^7^Li NMR peaks are mainly observed at approximately 0 and 250 ppm. The resonance around 250 ppm can be ascribed to lithium metal in the anode of the battery because this signal intensity depends little on the progress of the battery reactions (Figure S8c) [39]. On the other hand, the peak around 0 ppm is ascribed to Li^+^ ions in the electrolyte and close to the POM molecule in the cathode [40,41]. As shown in Figure 5a, the chemical shift of the main signal around 0 ppm depends little on the discharge processes despite the reduction of Mo ions. This absence of paramagnetic shifts suggests that paramagnetic interactions between the ^7^Li nuclei and Mo ions are rather weak in these samples, presumably because the lithium ions are effectively shielded from the oxygen ligands of the POM clusters. 

On the other hand, the intensity of the peak around 0 ppm was reversibly changed during charge/discharge; Li^+^ ions from the Li metal anode moved to the cathode, and this peak intensity ascribed to Li^+^ ions increased during discharge, while a reversible reaction occurred in a charge process. Therefore, the intensity evolution of the main peak around 0 ppm was described with the battery voltage change (Figure 5b). This intensity increase in discharge implies that a large amount of Li^+^ ions is adsorbed to the cathodes (i.e., nanohybrid materials), and EDL is formed in the discharge process. In contrast, the intensity decreases in the charge process, indicating that Li^+^ ions move far from POM clusters and EDL disappears. We compared this intensity evolution with those of ^7^Li NMR in LIBs using only the C320 carbon and only POM during charge/discharge. Figure 5b suggests a significantly high intensity increase in the discharge for the POM/C320 nanohybrid materials, indicating that more Li^+^ ions are adsorbed onto their surfaces and a larger EDL is formed, compared to those for C320 and POM. This implies that the nanohybridisation of POM with porous carbon can provide a functional surface in which a large EDL is formed, leading to high-performance supercapacitance.

## 4. Conclusions

We prepared nanohybrid materials of POM/CMK-3, POM/C320, POM/C500 and POM/C642, in which individual POM molecules are loaded inside of the porous carbon, as cathode materials for LIBs. During charge–discharge, the POM/C320-MCB exhibited a higher battery capacity and stable cycle retention at a high rate, compared to the other POM/carbon-MCBs as in summarised in Table 2. In situ XAFS and ^7^Li solid-state NMR analyses of C320/POM indicated the coexistence of capacitor effects of C320 and valence change of POM in the C320/POM nanohybrid materials. The increased cycle performances and capacities were owing to the fascinating architecture of C320 with a 3D structure, a suitable pore size, and the highest surface area among the carbons used in this work. This work highlights the excellent performance of porous carbon with a three-dimensionally linked pore network structure as an additive for supercapacitors to realise high-performance energy storage devices.

## Figures and Tables

**Table 1:** Brunauer–Emmett–Teller (BET)-specific surface area, structure and pore size of mesoporous carbons.

Mesoporous Carbons	Area/m^2^g^−1^	Pore Size	Structure
C320	1430	2.1 nm	3D
CMK-3	930	3.8 nm	1D
C642	690	<2 nm	3D
C500	490	<2 nm	3D

**Table 2:** Electrochemical performances of POM-based electrode materials used in lithium ion batteries.

Electrode Materials	Initial Discharge Capacities	Rate Performance	Cycling Stability	Reference
TBA_3_[PMo_12_O_40_]/C320	489 mA h g^−1^@50 mA g^−1^	480 mA h g^−1^@50 mA g^−1^	98%, 10 cycles@1 A g^−1^	This work
TBA_3_[PMo_12_O_40_]/SWNT	320 mA h g^−1^@1 mA g^−1^	N/A	94%, 10 cycles@1 A g^−1^	[30]
TBA_3_[PMo_12_O_40_]/RGO	140 mA h g^−1^@1 mA g^−1^	183mA h g^−1^@1 mA 105mA h g^−1^@2 mA 75mA h g^−1^@4 mA	98%, 10 cycles@1 A g^−1^ 98%, 10 cycles@2 A g^−1^ 95%, 10 cycles@4 A g^−1^	[31]
Li_7_[V_15_O_36_(CO_3_)]	250 mA h g^−1^@50 mA g^−1^	170 mA h g^−1^@2 mA g^−1^ 140 mA h g^−1^@10 mA g^−1^	82%, 100 cycles@2 A g^−1^	[42]
Mo-V-Bi-O	320 mA h g^−1^@1 mA g^−1^	N/A	65%, 20 cycles@1 A g^−1^	[43]
PANI/PMo_12_ nanofiber	183.4 mA h g^−1^@0.1 mA g^−1^	183mA h g^−1^@0.1 mA g^−1^ 94mA h g^−1^@2 mA g^−1^	81%, 50 cycles@0.1 A g^−1^	[44]
[H_2_Metf]_3_[V_10_O_28_]	156 mA h g^−1^@1 mA g^−1^	N/A	64%, 20 cycles@1 A g^−1^	[45]
Mn_3_V_19_-HIL/RGO	214 mA h g^−1^@100 mA g^−1^	210 mA h g^−1^@100 mA g^−1^ 121 mA h g^−1^@5 mA g^−1^	89%, 100 cycles@100 A g^−1^	[46]
NH_4_V_3_O_8_ microbelts	201mA h g^−1^@90 mA g^−1^	201 mA h g^−1^@90 mA g^−1^	90%, 100 cycles@100 A g^−1^	[47]

## Supplementary Materials

The following are available online at https://www.mdpi.com/1996-1944/13/1/81/s1, Figure S1: EDX spectrum of C320/POM hybrid materials, Figure S2: IR spectra of the (a) POM, C320 and POM/C320 hybrid materials, (b) POM, C500 and POM/C500 hybrid materials, (c) POM, C642 and POM/ C642 hybrid materials, (d) POM, CMK-3 and POM/CMK-3 hybrid materials, Figure S3: Charge–discharge curves of (a) CMK-3, (b) C320, (c) C500 and (d) C642 in the voltage range of 1.5–4.2 V with a constant current of I = 1.0, Figure S4: Charge–discharge curves of (a) POM/CMK-3, (b) POM/C320, (c) POM/C500, (d) POM/C642 corresponding respectively to CMK-3, C320, C500 and C642 in the voltage range of 1.5–4.2 V with a constant current of I = 1.0 mA, Figure S5: (a) The first discharge curves of MCBs including C320/POM nanohybrid materials with various ratio in the cathode, (b) Cycle performances of MCBs including C320/POM nanohybrid materials with various ratio in the cathode, Figure S6: (a) The first charge/discharge curves of carbon black, (b) Cycle performances of carbon black, Figure S7: Operando Mo K-edge XANES spectra for the POM/C320 MCBs in the first discharge process, Figure S8: (a) Charge–discharge curves of in situ solid-state 7Li NMR POM/C320-MCBs. (b) Full range of solid-state 7Li spectrum of POM/C320-MCBs. (c) High-resolution 7Li NMR spectra for 1C and 1D at approximately 255 ppm.
