# Supplementary material for: Preparation and Carbon-Dependent Supercapacitive Behaviour of Nanohybrid Materials between Polyoxometalate and Porous Carbon Derived from Zeolitic Templates
Source: Materials (Basel). 2019 Dec 22;13(1):81. doi: 10.3390/ma13010081 (PMC6982092; doi:10.3390/ma13010081)
Supplement: Supplementary file 1 [file materials-13-00081-s001.docx]

Supplementary Materials

Preparation and Carbon-Dependent Supercapacitive Behaviour of Nanohybrid Materials Between Polyoxometalate and Porous Carbon Derived from Zeolitic Templates

Heng Wang, Takeshi Shimizu and Hirofumi Yoshikawa


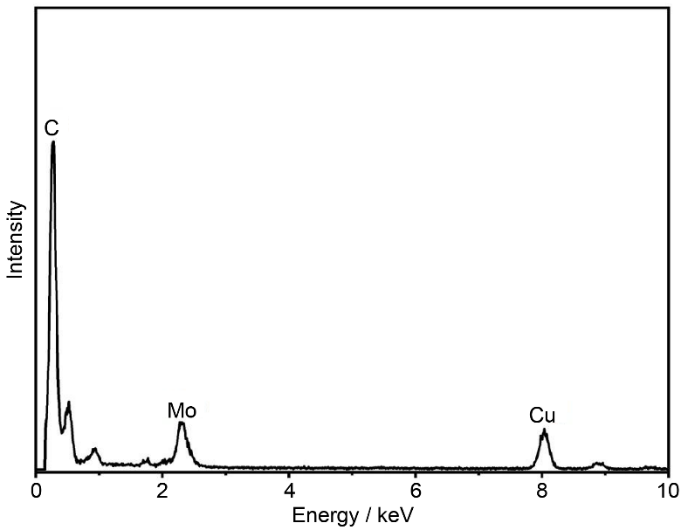


**Figure S1.** EDX spectrum of C320/POM hybrid materials.


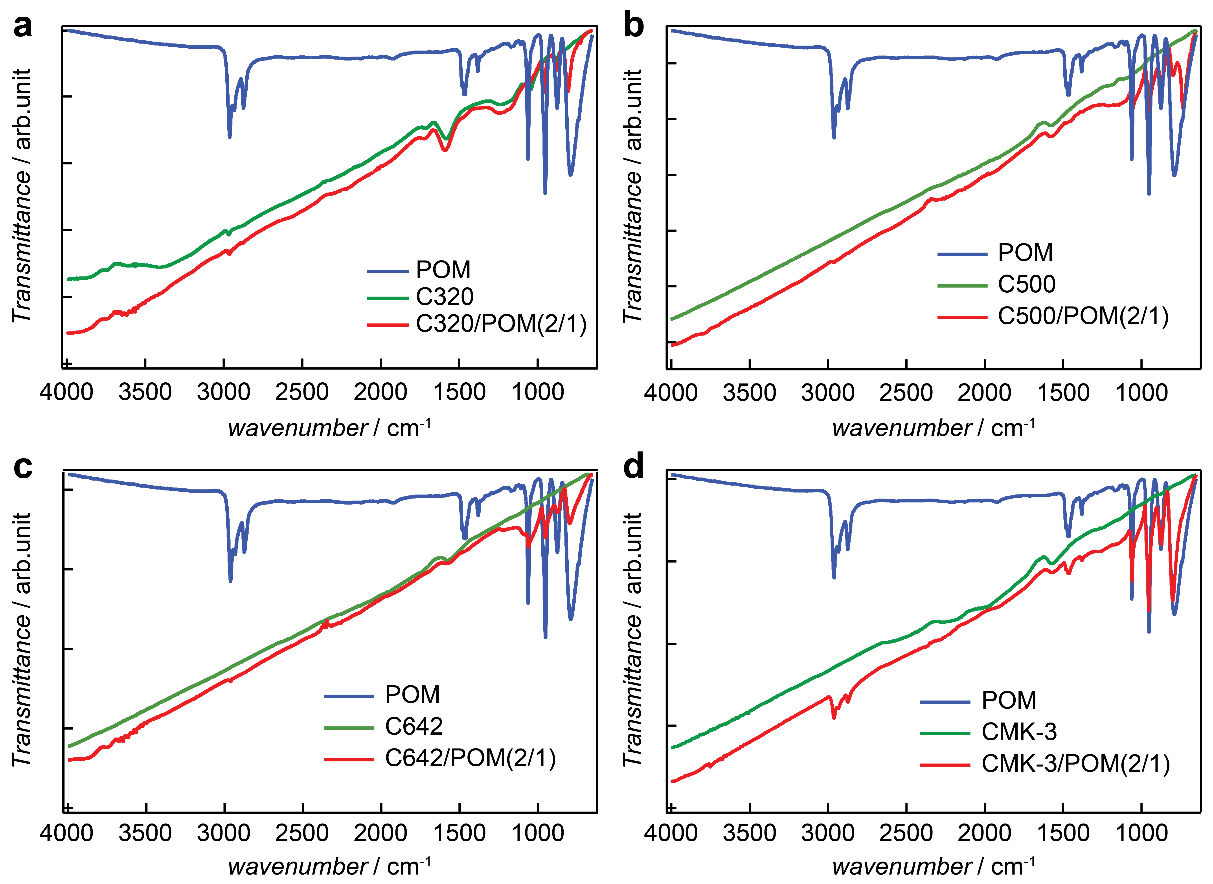


**Figure S2.** IR spectra of the (a) POM, C320, and POM/C320 hybrid materials, (b) POM, C500, and POM/C500 hybrid materials, (c) POM, C642, and POM/ C642 hybrid materials, (d) POM, CMK-3, and POM/CMK-3 hybrid materials.


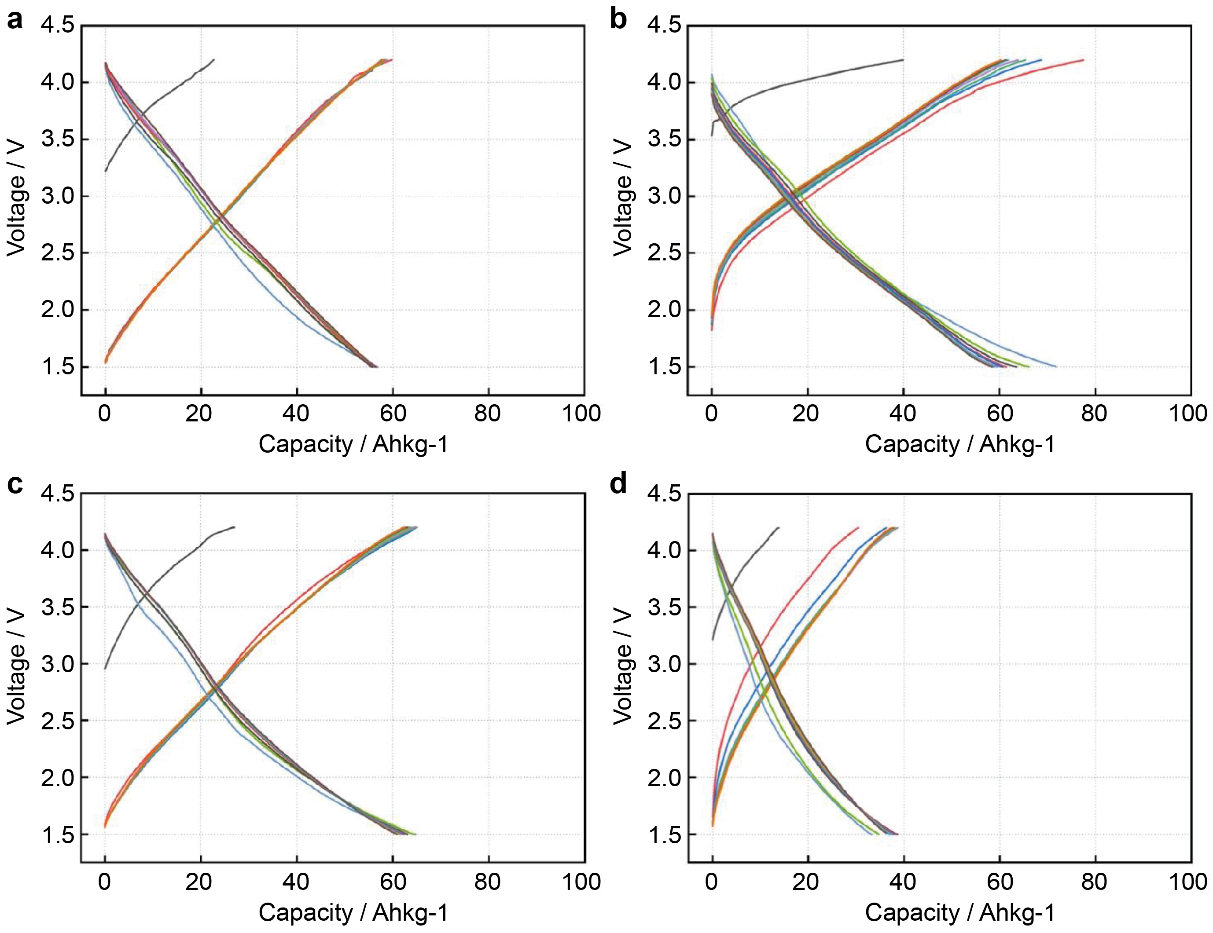


**Figure S3.** Charge–discharge curves of (a) CMK-3, (b) C320, (c) C500, and (d) C642 in the voltage range of 1.5 - 4.2 V with a constant current of *I* = 1.0 mA.


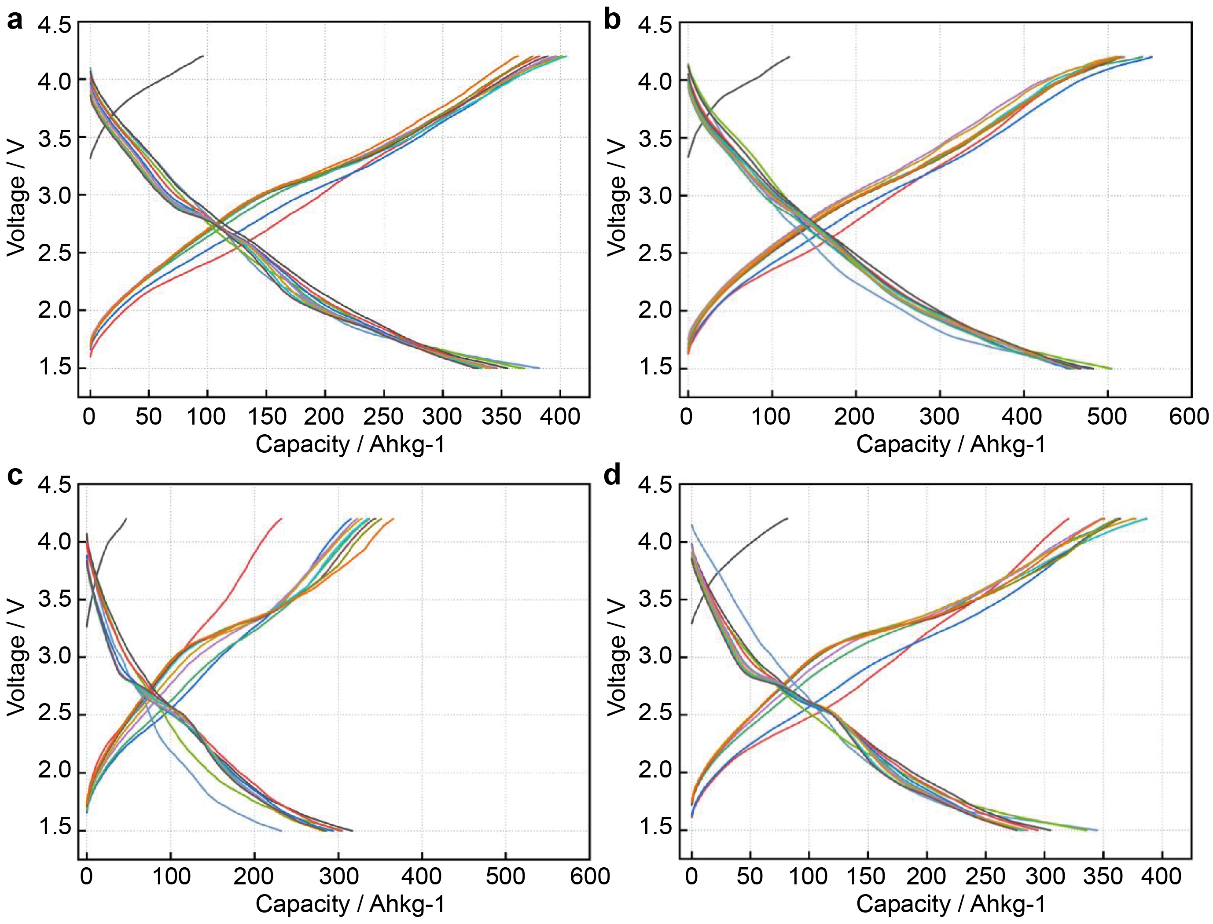


**Figure S4.** Charge–discharge curves of (a) POM/CMK-3, (b) POM/C320, (c) POM/C500, (d) POM/C642 corresponding respectively to CMK-3, C320, C500, and C642 in the voltage range of 1.5 - 4.2 V with a constant current of *I* = 1.0 mA.


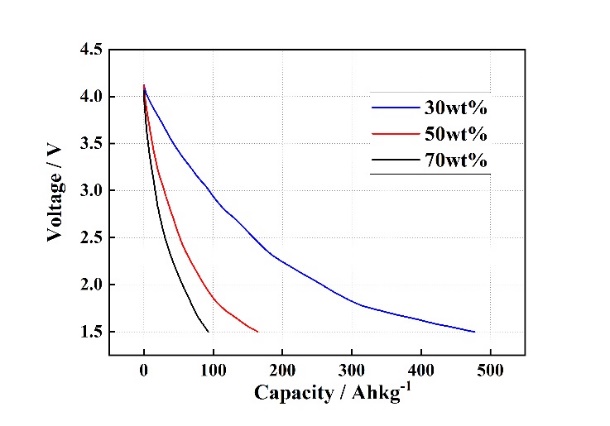

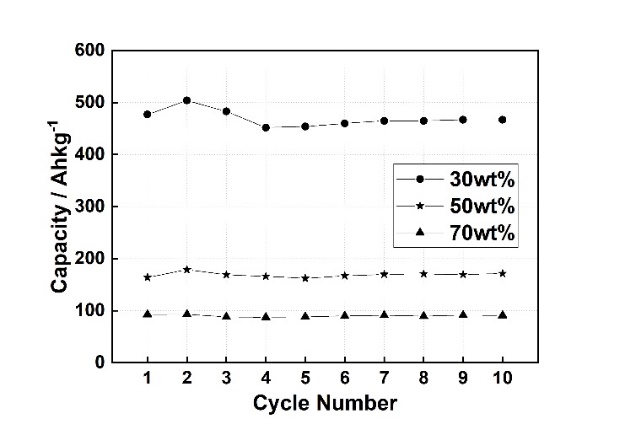


(b)

(a)

**Figure S5.** (a) The first discharge curves of MCBs including C320/POM nanohybrid materials with various ratio in the cathode, (b) Cycle performances of MCBs including C320/POM nanohybrid materials with various ratio in the cathode.


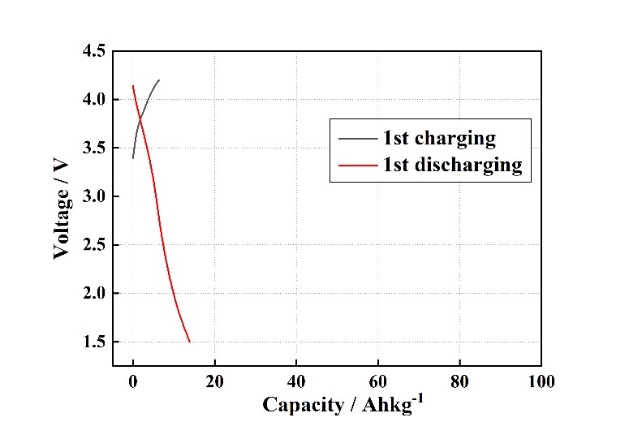

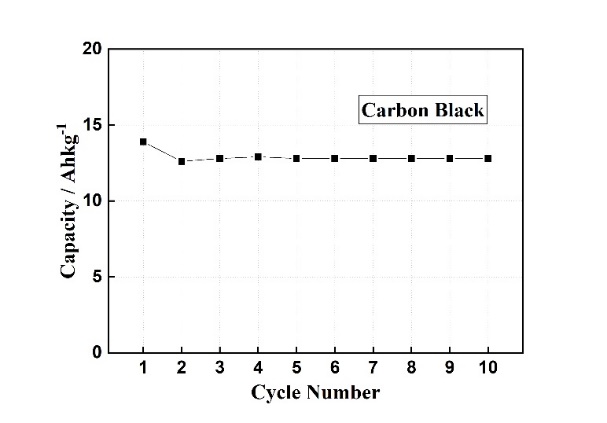


(a)

(b)

**Figure S6.** (a) The first charge/discharge curves of carbon black, (b) Cycle performances of carbon black.


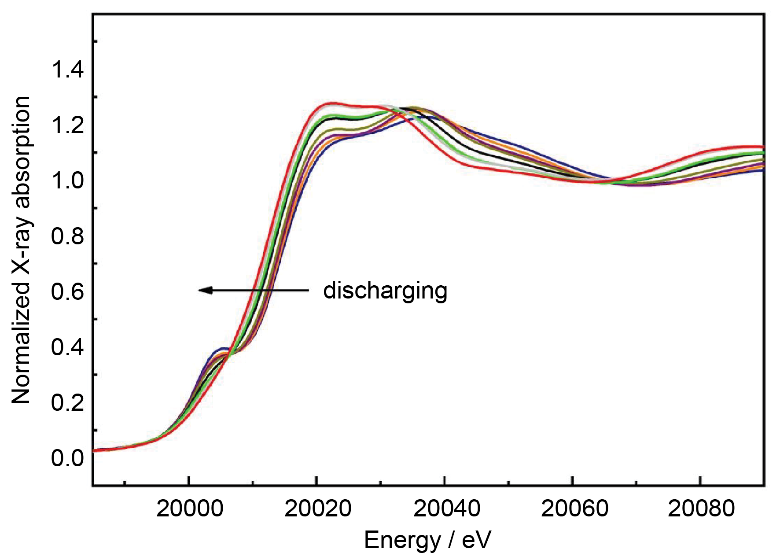


**Figure 7.** Operando Mo *K*-edge XANES spectra for the POM/C320 MCBs in the first discharge process.


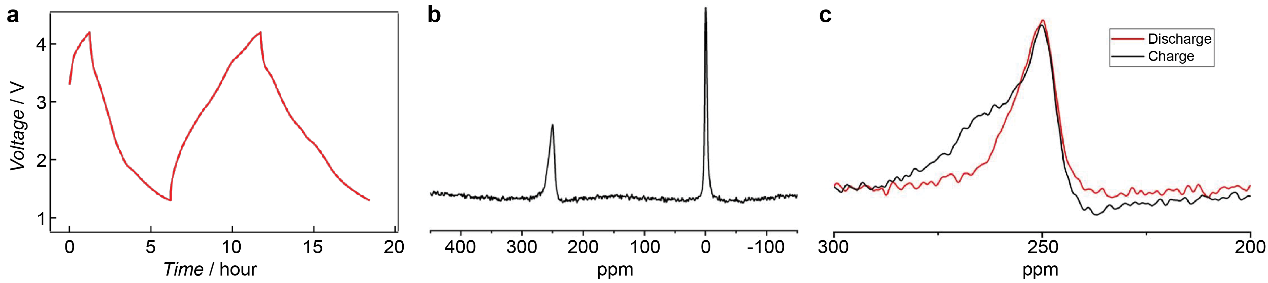


**Figure S8.** (a) Charge–discharge curves of in situ solid-state ^7^Li NMR POM/C320-MCBs. (b) Full range of solid-state ^7^Li spectrum of POM/C320-MCBs. (c) High-resolution ^7^Li NMR spectra for 1C and 1D at approximately 255 ppm.

| 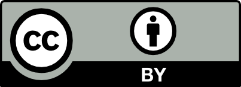 | © 2020 by the authors. Submitted for possible open access publication under the terms and conditions of the Creative Commons Attribution (CC BY) license (http://creativecommons.org/licenses/by/4.0/). |
| --- | --- |
